# A Primary Gastrointestinal Presentation and Novel Genetic Variant of Dyskeratosis Congenita in a Pediatric Patient

**DOI:** 10.1097/PG9.0000000000000242

**Published:** 2022-08-16

**Authors:** Jeffrey Lee, Edward Cheeseman, Maria Matheus, Nagraj Kasi

**Affiliations:** From the * Division of Pediatric Gastroenterology, Hepatology, and Nutrition, MUSC, Charleston, SC; † Division of Pediatric Ophthalmology, Medical University of South Carolina, Charleston, SC; ‡ Division of Neuroradiology, Medical University of South Carolina, Charleston, SC.

## Abstract

Dyskeratosis congenita (DC) is a rare telomerase disorder affecting high turnover cells. Malfunction of protective proteins in DC results in patient genomes with shortened germline telomeres leading to genetic instability, cellular apoptosis, and overall cellular lifespan degradation. Classically, reports of DC described a triad of dysplastic nails, reticular skin pigmentation, and oral leukoplakia. However, more recent reports have focused on disease presentation affecting other high turnover organ systems including the gastrointestinal system. Patients may present with dysphagia because of esophageal stricture/web, diarrhea secondary to enteropathy or enterocolitis. We present a pediatric patient who presented with feeding difficulty secondary to an esophageal stricture as the primary manifestation of DC. She was diagnosed with Revesz Syndrome, a rare subtype of DC, along with a novel genetic variant not previously reported. This report serves to bring awareness to gastroenterologists that DC, though classically thought to present with dermatological findings, can present with primary gastrointestinal manifestations.

## INTRODUCTION

Dyskeratosis congenita (DC) is a multisystem disorder resulting in patients with very shortened germline telomeres (1). Telomeres are nucleotide repeats at chromosomal ends, which maintain chromosomal integrity. Telomeres naturally shorten with each cell division causing attrition over time. Enzymes and protein complexes such as telomerase, shelterin, and dyskerin aid in maintaining telomere length and integrity. Failure of this leads to genetic instability/degradation, cellular apoptosis, and overall decreased cellular lifespan. DC typically affects organs with high cellular turnover including the dermatological, hematological, and gastrointestinal systems and the most severe sequelae include bone marrow failure (BMF), leukemia, or solid tumors.

DC was first described primarily with description of dermatological manifestations including a classical triad of dysplastic nails, reticular pigmentation of the neck and back, and oral leukoplakia. As more cases were reported, other organ manifestations were discovered and described. However, diagnostic criteria continues to require one of the above dermatological manifestations to meet diagnosis. To date, 11 genetic pathogenic variants have been identified. Depending on the genetic variant, inheritance pattern can be X-linked, autosomal recessive, or autosomal dominant (1).

## CASE REPORT

A 16-month-old female was referred to our gastroenterology clinic with poor feeding. Parents reported gastroesophageal reflux symptoms and vomiting since transitioning from liquid to solid foods at 6 months of age. She was born at 37 weeks and was small for gestational age (birth weight of 1665 g). Because of abnormal Doppler readings, oligohydramnios with intrauterine growth restriction, and low biophysical profile, she was delivered via cesarean section.

**FIGURE 1.**
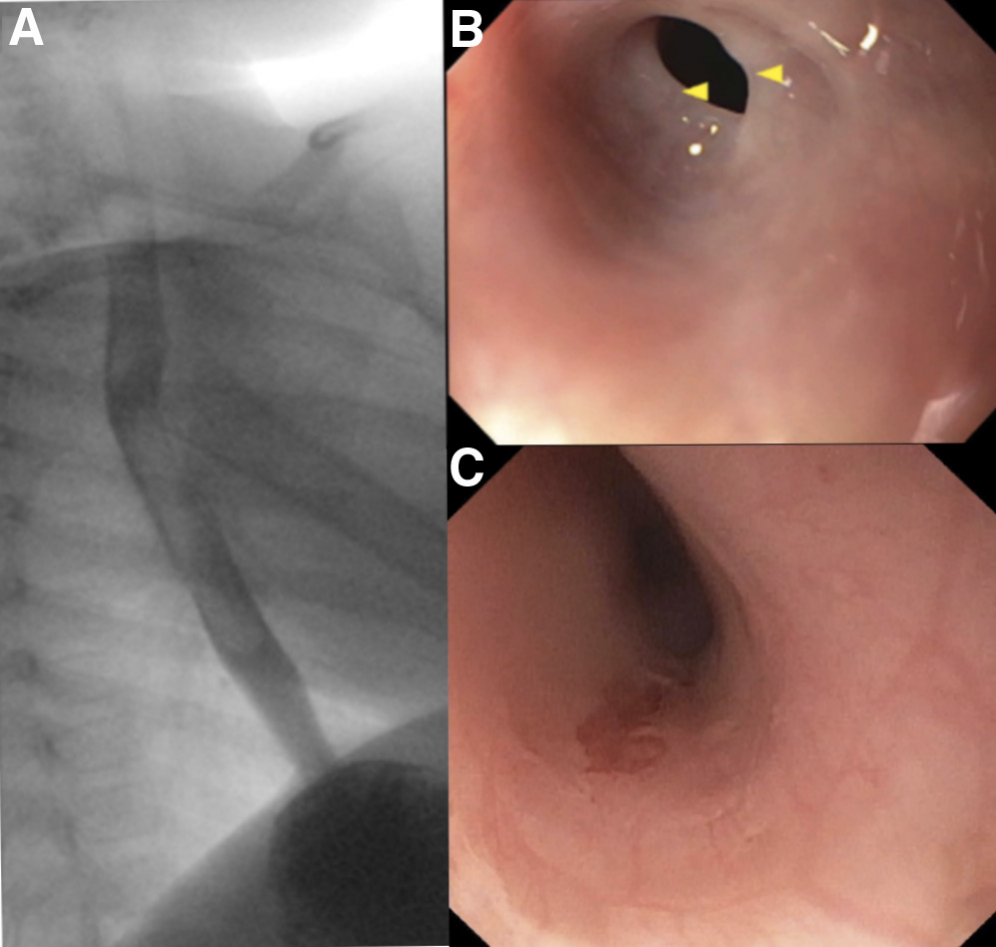
(A) Normal lateral view on esophagram. (B) Esophageal stricture predilation. (C) Esophageal stricture postdilation.

**FIGURE 2.**
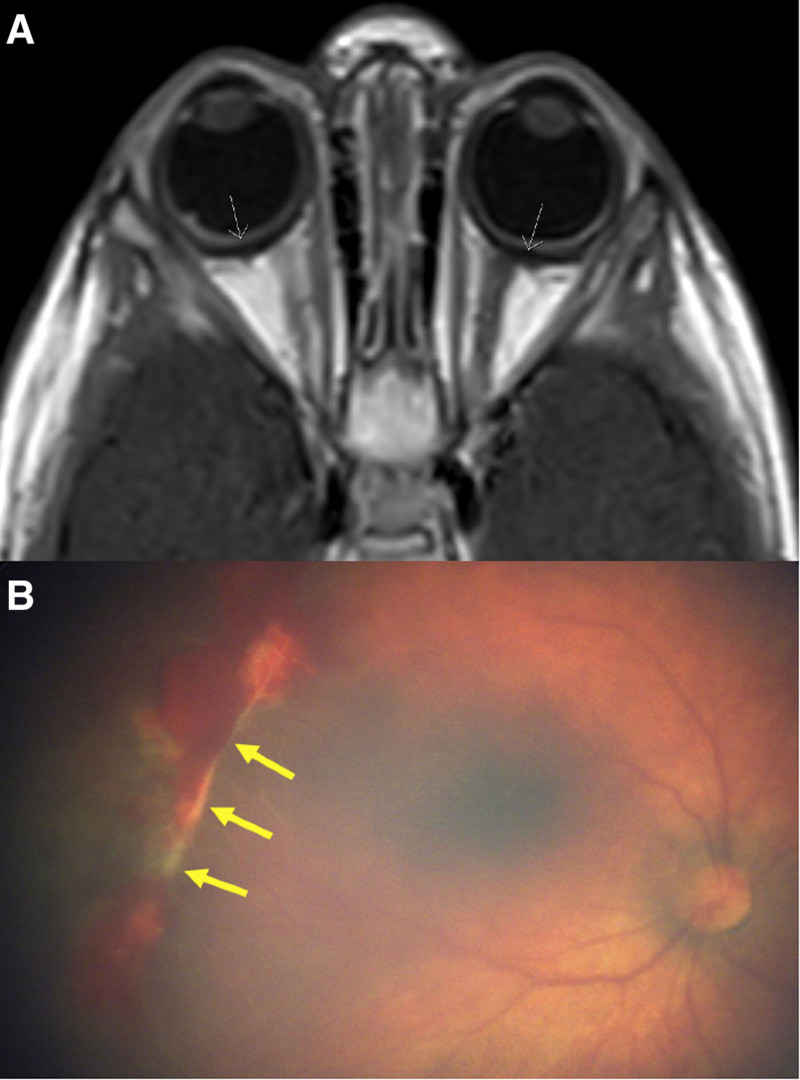
(A) Retinal exudate (arrows) seen on magnetic resonance imaging of the brain. (B) Retinal exudate (arrows) seen on standard ophthalmological exam.

Apart from poor weight, her systemic exam was normal. At 13 months of age, she underwent an initial work-up of laryngoscopy and modified barium swallow study which was unremarkable. An esophagram also performed 1 month later was read as “difficult and limited but normal esophagram” (Fig. 1A). She underwent various feeding therapies at the time without any significant improvement in symptoms, and her pediatrician had prescribed acid suppression therapy which did not seem to improve her symptoms. Because of continued symptoms, at 17 months of age, she underwent a combined ENT, pulmonary and gastrointestinal procedures of microlaryngoscopy, bronchoscopy, and upper endoscopy together. The former two procedures were unremarkable; however, endoscopy revealed a benign proximal esophageal stricture with an internal diameter of approximately 4–5 mm and 1 mm in length (Fig. 2). She was dilated with 18 to 21 Fr bougie dilators sequentially without complication including bleeding or perforation. Of note, physical exam on the day of her procedures was significant for new pallor not previously noted but no other dermatological abnormalities thus a complete blood count (CBC) was ordered. This revealed severe pancytopenia (white blood count 2.4 k/µL, hemoglobin 8 g/dL, hematocrit 23%, platelets 22 k/µL). Upon chart review, the patient was being followed by the pediatrician for an isolated mild anemia and borderline thrombocytopenia discovered on routine testing at her one year well child exam (hemoglobin 10.5 g/dL, hematocrit 29.3%, platelets 140 k/µL). A follow-up blood count performed by the pediatrician 1 month before her presentation revealed mild improvement in her anemia (hemoglobin 11.1 g/dL, hematocrit 30.7%) without intervention but now platelets were noted to be low at 46 k/µL. This was attributed to be a laboratory error because of the sample clotting. By the time, laboratory results drawn at her endoscopic procedure resulted the patient had already been discharged home thus no blood product was given but she was urgently referred to pediatric hematology. As part of their evaluation, a bone marrow biopsy was performed which revealed megakaryocyte hypoplasia. A BMF syndrome panel and telomere analysis was performed. BMF panel revealed a heterozygous pathogenic nonsense variant in the *TINF2* gene, c.841C>T (p.Glu281*). Telomere analysis revealed extremely short telomeres in all lymphocytes, granulocytes, natural killer cells, all below the first percentile. Magnetic resonance imaging of the brain revealed bilateral globe thickening consistent with retinal exudate (Fig. 1B). Ophthalmology exam revealed exudative proliferative retinopathy bilaterally (Fig. 1C) requiring laser photocoagulation and anti-vascular endothelial growth factor therapy. Based on clinical and genetic findings, she was diagnosed with Revesz syndrome and listed for hematopoietic cell transplant (HCT).

Although awaiting transplant, because of recurrence of difficult feeding, she underwent repeat upper endoscopy revealing stricture recurrence requiring balloon dilation twice with improvement in symptoms. Six months after the diagnosis of Revesz syndrome, she underwent a successful HCT. At 1-year post transplant, there was no recurrence of esophageal stricture. Her feeding difficulties have resolved and she is thriving.

## DISCUSSION

Although classic presentation has emphasized dermatological findings, other organ systems may present as the primary presentation of DC. A wide range of primary gastrointestinal manifestations have been reported including: esophageal stenosis (most common), enteropathy with diarrhea and poor growth (second most common), noncirrhotic pulmonary hypertension, hepatopulmonary syndrome, vascular ectasias of the gastrointestinal tract resulting in bleeding, anorectal adenocarcinomas, and anal strictures (2–6). In addition, an institutional evaluation of their telomere syndrome registry by Joussaint et al (2) found that 16% of patients had gastrointestinal involvement and, often times, dysphagia or diarrhea were the sole symptoms at presentation.

In addition to her diagnosis of DC, the patient was found to have a more severe variant called Revesz syndrome that, as of publication of this report, has only been reported 18 times in the English literature. It is associated with even shorter telomeres than classical DC and patients can also present with intracranial calcifications and bilateral exudative retinopathy which she had on radiological and ophthalmological exam (Fig. 2A,B) (7). In this particular case, patient was also found to have a novel mutation variant of her *TINF2* gene never published before.

Treatment of DC is typically individually tailored and primarily supportive management (1). In the case of esophageal stenosis, many patients require repeat dilation, whereas those with enteropathy may need colectomy or dependence on parental nutrition. For patients with bone marrow failure or leukemia, hematopoietic cellular transplant (HCT) may be required. In our case, patient tremendously benefited from HCT.

DC can present solely with gastrointestinal symptoms and, in our case, without any dermatological findings. In our case, the patient’s mild anemia and thrombocytopenia was not considered as part of her disease and her esophagram, although limited in evaluation, did not identify her stricture. It was only after more comprehensive and invasive evaluation that the patient’s rare and severe phenotype of DC was diagnosed. Thus, a disease such as DC can be missed if only the classical triad is considered. We hope that this case brings awareness to gastroenterologists regarding nonclassical, primary gastrointestinal presentations of DC.

## ACKNOWLEDGMENTS

Jeffrey Lee drafted the manuscript, Edward Cheeseman and Maria Matheus provided imaging, and Nagraj Kasi edited and approved the final draft submitted.
